#  Effect of Whole Exome Sequencing in Diagnosis of Inborn Errors of Metabolism and Neurogenetic Disorders

**Published:** 2018

**Authors:** Marjan SHAKIBA, Mohammad KERAMATIPOUR

**Affiliations:** 1 Departmentof pediatric Endocrinology and Metabolism, Mofid Children’s hospital, Shahid Beheshti university of medical sciences, Tehran, Iran.; 2 Department of Medical Genetics Faculty of Medicine, Tehran University of Medical Sciences,Tehran, Iran.

**Keywords:** Whole exome sequencing, Next-generation sequencing, Metabolic disease, Neurometabolic disease, Neurogenetic disease, Inborn errors of metabolism

## Abstract

**Objective:**

Inborn errors of metabolism are complex disorders with huge variability in clinical manifestations. Decreasing cost of whole exome sequencing (WES) in recent years, made it affordable. Therefore, we witnessed an increase in using WES in diagnosis of genetic diseases, including inherited metabolic disorders.

**Methods:**

A systematic search was done in well-known databases including Medline, Google, Cochrane, and PubMed until 1 Oct 2017. We reviewed the articles addressing the use of WES in diagnosis of metabolic and neurogenetic diseases to evaluate its impact in diagnosis of these conditions.

**Results:**

WES is an effective technology with remarkable impact in diagnosis of metabolic and neurologic diseases, especially in complex cases. Diagnostic yield of WES for these conditions has large variety, ranging from 16% to 68% with an increase during recent years. WES can provide fresh valuable information about new disease, new variants and phenotypes. Careful analysis and interpretation of data obtained by WES and precise evaluation of correlation between clinical manifestation and WES findings are necessary to achieve a correct diagnosis.

**Conclusion:**

WES is effective and useful technology for diagnosis of metabolic and neurogenetic diseases, especially in complex or unsolved cases.

## Introduction

There is a growing rise in using whole exome sequencing (WES) in diagnosis of genetic diseases during recent years. Considering the decreasing cost of the technique, it is becoming a routine step in diagnostic approaches for any unresolved clinical problem. This trend in using WES in clinics creates both opportunities as well as challenges (1). 

Therefore, it is very important for physicians and patients to be aware of different aspects of using WES as a diagnostic tool and have information about factors that effect on efficacy of whole exome sequencing. 

Due to huge overlap of clinical manifestations and biochemical findings in neurogenetic and inborn errors of metabolism, clinical and biochemical evaluations are inconclusive in many situations. On the other hand, neuronal tissues are not easily accessible for pathologic evaluations (2). Therefore, clinicians face with a large list of differential diagnosis that makes difficulty in finding accurate diagnosis for suspected metabolic or neurogenetic cases.

Using traditional methods such as Sanger sequencing for genetic investigation of these disorders proved to have limited results with huge cost (2). Many of these disorders have allelic as well as locus heterogeneity with no major allele or locus responsible in significant fraction of patients. Therefore, a targeted approach with analysis of a small genomic region has no benefit for majority of cases. Such limitations of Sanger sequencing were addressed successfully by introduction of Next Generation Sequencing (NGS) that made the high throughput sequencing of DNA a reality. Having NGS in our hand, we are able to sequence as many genomic regions as we wish, even the completely human genome. Still, sequencing of completely human genome is not the choice option for many cases in a clinical setting, due to the many pitfalls in analysis and interpretation stage as well as relatively high cost.

On the other hand, more than 80% of disease-causing variants are located in or adjacent to exons that contain the coding regions of human genome (3-4). Therefore, sequencing of exons and their flanking regions seems a very reasonable choice for clinical purposes. So WES that covers all known exons and their flanking regions became the method of choice for genetic analysis of many complex metabolic and neurogenetic cases. 

There are many studies with focus on the role of WES in diagnosis of metabolic and neurogenetic disorders; here we tried to offer a comprehensive review of such published studies. 

## Materials & Methods

This search was accomplished to identify articles in which the role of WES and next-generation sequencing in diagnosis of metabolic and genetic disorders had been evaluated as a focus.

A systematic search was done in well-known databases including Medline, Google, Cochrane, and PubMed until 1 Oct 2017**. **The search included keywords as follows: WES, next-generation sequencing, inborn error of metabolism, metabolic disorders, neurometabolic disorders, neurogenetic disorders, genetic disorders and diagnosis. Abstracts were reviewed to screen articles that were relevant to our goal. In addition, reference lists of articles were screen to find other possible studies that were relevant to our subjects and several articles returned to add to our lists. Only studies were chosen published in peer-reviewed English journals. After surveying studies, article was written regarding the subject in narrative format. 

**The role of WES in diagnosis of inborn errors of metabolism and neurogenetic disorders**

Application of WES and next-generation sequencing as a diagnostic tool is a revolution in diagnosis of puzzling and complex diseases in which biochemical studies and other paraclinical investigations have not resulted in diagnosis. WES is employed to diagnose different genetic diseases more and more with regard to decreasing in cost of test. Therefore, it is very important for health professionals to be aware of different aspects of the technique including its advantages and limitations to have a rational expectation of the assay in regards to patient management and follow up.

We tried to answer important questions regarding the role of WES in diagnosis and management of inborn errors of metabolism and neurogenetic diseases:

How effective is WES in diagnosis of unknown and puzzling diseases?

How much can WES effect on management of patients?

What are the factors that influence efficacy of WES in diagnosis of diseases?

What is the impact of WES in identifying new disease-causing genes?

What is the role of WES in identifying new clinical phenotype for known genes?

How effective is WES in defining diagnosis for patients who have blending phenotype? 

Dose WES increase our knowledge about disrupting/disease-causing variants?

What are challenges in application of WES as a routine diagnostic route?

Is WES cost effective for practicing in diagnostic field as a routine test?

Is incidental finding a challenge in WES? 

**How effective is WES, in diagnosis of unknown and puzzling diseases?**

It is essential for physicians, patients, and their families to know the success rate of WES in achieving a diagnosis for patients. Our review showed various reported success rates depending on the clinical presentation of the patients. 

In a study, patients with intellectual disability underwent WES by 87% mean exome coverage. It results in definite diagnosis in 16% of participants (5). 

 In a study, 119 trios with undiagnosed suspected genetic disease enrolled in research and underwent whole exome sequencing. The diagnosis was confirmed in 29 patients (24.4%) that consist of 45% de novo mutations (6).

 Another study was designed to discover inborn errors of metabolism in patients who have neurologic complain and their diagnosis remains unknown despite complete investigations. They combined clinical signs, symptoms, and biochemical finding carefully to use them in interpretation of test results. They found a genetic diagnosis in 68% of participants. They benefit collaboration between clinicians and bioinformaticians to interpret their results (7). 

A large study with 2000 participants was done on 1756 patients with neurological problems. This study resulted in diagnosis in 25.9% of patients with neurologic complaints and 25.2% positive results in all 2000 patients that confirmed previous results obtained (8, 9). 

WES is beneficial in routine practice for diagnosis of patients with genetic disease in their study (2). In 3 other studies, diagnostic rate of WES was reported 25%, 45% and 50.5% in patients with neurologic problems and suspected metabolic disorders (10-12). 

Another study was evaluated efficacy of clinical exome sequencing (CES) in undiagnosed patients. In CES, clinical phenotypes were applied to filter data for interpretation. They compared the rate of positive results in Trio-CES and proband-CES. They found diagnostic yield in Trio-CES was significantly higher than proband-CES, 31% against 21%. Total diagnostic yield in 814 studied cases was 26% whereas it was 28% in patients who complain of developmental delay (13). 

Overall, the tendency to utilize WES rise as a facility to diagnose mendelian disorders such as metabolic diseases (14). 

**How much can WES effects on management of patients?**

Patient management or disease management includes many actions related to the patient and the patient family including treatment, screening, prevention, and reproductive planning such as pre-implantation and prenatal genetic diagnosis. Different studies reported different changes in patient’s management based on the result or diagnosis obtained by WES. The least effect of definite diagnosis is the relief and better feeling for the patients and their families from burden of unknown diseases. 

About 44% of patients gained from genetic diagnosis. These benefits consist of change in treatment and preventive measures. Genetic diagnosis with change in treatment can affect patient’s outcome through the changes in treatment and provide measures for prenatal diagnosis in patent’s family (7). 

Presence of a defined diagnosis has a major role in facilitating making difficult decisions for high-risk treatments such as bone marrow transplantation (7).

Four patients (3.4%) underwent changes in their treatment following new diagnosis made by genetic analysis (6). Two percent rate of change in treatment was reported (5). On the other hand, 49% change was reported in management of their patient following the result of WES that also helped in understanding pathophysiology of diseases. In 10 patients (23%), changes were implemented at the level of drug and dietary treatment (10).

**What are the factors that influence efficacy of WES in diagnosis of diseases?**

Certainly, there are many elements in the laboratory or analytical step of applying WES in genetic diagnosis affect the outcome of the test. Proper quality control measures must be in place from sample preparation step to data analysis and report to ensure a reliable outcome. High-quality DNA is essential for a successful NGS experiment. Available whole exome capturing systems have different capabilities in enrichment of target exons before entering the sequencing step. Sequencing platforms have different specification in terms of data output and sequencing error rates. Available bioinformatics pipelines for data analysis have different characteristics. Above all, variant interpretation itself is a complex issue. The reliable outcome of WES is not reachable unless proper standards and guidelines are forced for all steps. Having all these in place, still getting different or even conflicting results for the same sample from different centers is unexpected. 

Assuming all steps in a WES experiment are performed with high standards, to ensure obtaining high-quality data, analysis and interpretation of the data itself are one of the most important as well as challenging part of test. WES provides massive amount of data, consist of tens of thousands of variants scattered among more than 20000 genes. These variants require a comprehensive annotation using available databases followed by a very careful filtering process to reach a small number of meaningful and desirable variants (15). Standards, guidelines, clinical and biochemical information must be considered in analyzing data (16). 

Close collaboration between bioinformaticians, geneticists, subspecialist clinicians, patients and patient’s family are essential for careful data analysis. Engaging basic scientists also can help in interpretation of result by finding relevancy between finding the genes and the variants, related biological pathways and molecular mechanism, or findings in model organisms with patient phenotype (7). Careful evaluation and interpretation of the variants in regards to their pathogenicity is a very important matter that influences the accuracy of outcome (6). Applying clinical data and using medical diagnostic software to filter data amplify data analyzing (8). The nature of clinical problems may influence in rate of positive results in WES (8). Some clinical problems are associated with higher diagnostic rate in WES (7, 10, 13). 

It is very important to be aware of the limitations of WES in analysis of some genomic regions due to the nature of sequence structure. WES is not able to detect some types of genomic variations, including large insertion/deletions, copy number variations, repeats expansions, deep intronic variants, and variants on mitochondrial genome (17).

**What is the impact of WES in identifying new disease-causing genes?**

A significant rise in identifying new disease-causing genes was witnessed following the introduction of NGS/WES. There are many reports showing the ability of WES in identifying new disease-causing genes and even providing treatment for patients based on defined pathophysiology of the disease following identifying the causative gene (7,15, 18-19). The ability to find new disease-causing genes is a major advantage of using WES compared to targeted gene panels for genetic investigation of patients with suspected genetic conditions. 

Two new inborn error of metabolism were found and 9-candidate gene could be responsible for intellectual disability. Identifying two candidate gene result in new treatment for patients (7). In another study the rate of finding new disease-causing gene estimated 3.4% (6). For identifying new disease-causing genes; the rate of new gene detection reported 8.5% (2).

**Table 1:** Comparison of studies that used whole exome sequencing for diagnosis of patients with neurologic problems and suspected metabolic disorders

Studies	M. Tarailo-Graovac’s Study (2016) (7)	Al-Shamsi’s study (2016) (11)	Zhu’s Study (2015) (6)	Yang’s Study (2014) (8)	Soden’ study (2014) (10)	Lee;s study (2014) (13)	Yang’s study (2013) (9)	Salazar’s study (2012) (2)	de Ligt,’s study (2012) (5)	
Participant’s number	41	85	119	1756	100	298	250	118		100	
Number of positive results	28(68%)	43(50.5%)	29(24.4%)	455(25.9%)	45(45%)	83(28%)	62( 25%)	40(33.9%)		16(16%)	
Suspected known disease		12(14%)	21(17.6%)								
Novel variants	53%	34%		57.8%							
Two coexisting disease	5(12%)		-	23(1.4%)			4(1.6%)				
Newly identified gene(Not previously associated with human diseases)	2(4.8%)		-		3(3%)					3(3%)	
Candidate gene (Not previously associated with human diseases)	9(21.9%)		4(3.4%)	67(12.9%)	9(9%)			22(18.6%)		21(21%)	
New phenotype for known gene	22(53%)		-		1(1%)	1(0.3%)	3(1.2%)				
Incidental findings	1(2.4%)			95(4.6%)		5%					
Mean coverage of exome		95%	94%	94.5%	80	93	95	96		87	
Depth of coverage		>20	>10	>20	>30	>10	> 20	>10		>10	

Overall, 24 candidate gene identified by exome sequencing in 100 patients affected by intellectual disability. For three of these candidate genes, the causative effects were confirmed by finding disruptive variants in the same genes in patients with similar problems (5). 

**What is the role of WES in identifying new clinical phenotype for known genes?**

 WES may find new phenotypes associated with previous known genes (7, 15, 18). Exome sequencing can also extend the spectrum of phenotypes result in a gene disruption (1, 13, 15, 20). 

**How effective is WES in defining diagnosis for patients who have blending phenotype?**

 Unusual combination of signs, symptoms, and biochemical phenotype sometimes can confuse even expert clinicians. This situation is soluble by using WES that discovers two or more coexisting genetic disorders. Similar experience reported a study that reported 5 of their probands (13.5%) suffered from two distinct monogenic disorders (7). Diagnosis of two distinct diseases in one patient was reported in 1.4 cases (4.6% of positive results) in a large cohort study (8).

**Dose WES help to increase our knowledge about disruptive/disease-causing variants?**

Many studies emphasizes on the role of WES in identifying new variants with disrupting effects on gene functions (1,2, 5, 7,8, 10, 21). Reporting such information is very helpful in interpretation and decision making when similar finding is obtained in the future for other patients with similar phenotype (22). 

**Is WES cost effective for practicing in diagnostic field as a routine test?**

Diagnostic approach to patients who complain of neurologic signs and symptoms often cost more than $ 10000 per patients (2). WES is very effective in reducing the cost of diagnostic approach for such patient. Performing various biochemical tests, or analysis of single candidate gene or panel of small numbers of genes before requesting WES, often increases the cost of diagnostic approach (1, 8). The cost of negative results test in difficult cases was estimated $19000 per family in comparison to $7640 for exome sequencing (10). The cost of WES is much less at this time, and it continues to decline, despite the relative unchanged cost of other para-clinical investigations. WES is cost effective as a diagnostic tool in clinic (15). Although this advantage should be weighed against some disadvantages of WES including patient privacy, undesirable incidental findings and so on. Next-generation sequencing continues to decline in cost (10).

**What are challenges in application of WES as a routine diagnostic method?**

Besides, the revolutionary effect of WES in diagnosis of genetic disorders, we should be aware of many limitations and challenges associated with using WES.

Occasionally, the genetic variant responsible for a disease may be missed in WES. This may be caused by poor capturing of the genomic region containing the variant. Limitations in sequencing step may also be the reason behind not detecting the causative variant. Sometimes, the causative variant remains unrecognized due to failure in the mapping or variant calling steps. 

WES only covers exons and their flanking regions. It has also limitations in detecting some variations based on the nature of the genomic variations. Therefore, causative variants outside the covered regions including intronic and non-coding regulatory regions cannot be detected. Variations such as structural genomic variants, uniparental disomy, large insertion/deletion/duplication, copy number variation, somatic mosaic variants, and variants on mitochondrial genome cannot be detected due to the nature of the technique employing wrong filters or not using appropriate filters for analyzing huge data result in missing right diagnosis (1, 8, 13, 23). Sometimes employing wrong filters or not using appropriate filters may result in missing right diagnosis. Complex inheritance patterns of the disease may be another reason that makes the diagnosis difficult (1). Another challenge in application of WES in clinic is recognizing clinically relevant variant among numerous variants of uncertain clinical significant (8, 23-26). Furthermore, more than 25% of disease-causing variants in available databases are incorrect. This makes variant interpretation very difficult (27). Accurate clinical and biochemical information is essential in many situations, to avoid misinterpretation of the result obtained by WES (15).

A defined diagnosis cannot be made due to the lack of enough knowledge about the function of candidate gene. In such cases, extensive functional studies may be required to prove causative relation between the candidate gene and variant and the patient clinical phenotype. 

**Is incidental finding a challenge in WES?**

In a cohort study of 92 patients, 5 cases of incidental findings were reported (4.6%), that all required medical intervention (8). In another study, the incidental actionable finding was reported in 12% of participants (9). Rate of incidental finding was reported 2.4% in another study (7).

Defining proper strategy in facing the incidental findings is an important challenge in WES. Although there are different opinions about reporting or not reporting such findings, all experts believe in necessity of setting appropriate guidelines regarding actions to be taken in such instances. Some authors recommend allowing individuals to have their own choices in regards to incidental findings (28).

## Discussion

Inborn errors of metabolism (IEM) are large spectrum of disorders with an excessive number of symptoms and signs that can affect all organ systems of human body. Clinical manifestations of these diseases are overlapping and it is very difficult to limit differential diagnosis only based on clinical findings. Inborn errors of metabolism are also among the differential diagnosis for other genetic diseases especially neurogenetic diseases due to having overlapping features.

IEM can be classified into three categories based on their diagnostic biomarkers. A number of IEM have specific diagnostic biomarkers. They do not need genetic analysis for confirmation of diagnosis. 

The second group of IEM has nonspecific biomarkers that allow clinicians to limit differential diagnosis but they are not distinctive enough to point a definite diagnosis. Using targeted next-generation sequencing covering appropriate panel of genes can be recommended genetic investigation in this group. 

The third group is metabolic disorders without known biomarkers. It is very difficult to list a limited number of diseases as possible diagnosis for these conditions. WES is the most cost-effective genetic investigation for this group.

Reviewing articles targeting efficacy of WES in identifying metabolic and neurogenetic disease argues efficacy of WES in identifying these disorders. There is considerable variety in rate of diagnosis, ranging from 16% to 68%. (1,2, 7, 5-15, 21, 29,30) (Table 1). Such wide range of success rate, were probably obtained due to differences in elements and steps of WES experiments in each study as well as the number of participants and the nature of their clinical problems. (9, 13). On the other hand, diagnostic yield is increasing over time, probably due to advances made in many elements of WES experiment, as well as improvements in data analysis and interpretation. Recent studies were reported higher diagnostic rate (7, 11). It is not rational to gathering the data of these studies for meta-analysis regarding reasons that mentioned above. In comparison, there are no notable differences in the rate of positive results when using targeted gene panels (20, 31,32). The ability to discover new disease-causing genes is the superiority of WES in comparison to targeted gene panels (2, 5-7, 10). On the other hand, WES can reduce the cost of diagnostic investigation by avoiding repeating tests, especially in puzzling cases, proving it a cost-effective diagnostic method in such cases (1, 2, 8, 10, 15). 

**In conclusion, **WES is an informative as well as cost-effective tool in research as well as in clinical settings. WES proved to be an effective research tool in identifying new genes and new diseases as well as defining new phenotypes or widening the spectrum of phenotypes resulted from deleterious variations in known genes. 

On the other hand, WES can significantly increase the diagnostic rate in clinical settings for metabolic and neurogenetic diseases. It can also significantly affect the patient management in terms of drug therapy and sometimes more complex interventions such as bone marrow transplantation. Therefore, it will not be long to see WES as a routine diagnostic test for many genetic conditions including metabolic and neurogenetic diseases. 

## Conflict of Interest

The authors declare that there is no conflict of interest.

## Author`s Contribution

Marjan Shakiba: Searching for the subject and screen articles, reviewing articles and choosing related ones, reading articles and gathering the results and writing review

Mohammad Keramatipour: Reading articles and writing review.
